# A phase 1 study to assess the absolute bioavailability, mass balance, pharmacokinetics, metabolism, and excretion of [^14^C]-mobocertinib, an oral inhibitor of EGFR exon 20 insertion mutations, in healthy participants

**DOI:** 10.1007/s10637-024-01446-y

**Published:** 2024-05-24

**Authors:** Michael J. Hanley, Steven Zhang, Robert Griffin, Sean Xiaochun Zhu, Robert J. Fram, Jianchang Lin, Karthik Venkatakrishnan, Neeraj Gupta

**Affiliations:** 1 Quantitative Clinical Pharmacology, Takeda Development Center Americas, Inc, Lexington, MA USA; 2 Global DMPK, Takeda Development Center Americas, Inc, Lexington, MA USA; 3 Clinical Science, Takeda Development Center Americas, Inc, Lexington, MA USA; 4 Statistical & Quantitative Sciences, Takeda Development Center Americas, Inc, Lexington, MA USA; 5 Quantitative Pharmacology, EMD Serono Research & Development Institute, Inc, Billerica, MA USA

**Keywords:** Mobocertinib, ADME, Tyrosine kinase inhibitor, Lung cancer, EGFR, Exon 20 insertion mutation

## Abstract

Mobocertinib (TAK-788) is a first-in-class oral epidermal growth factor receptor (EGFR) tyrosine kinase inhibitor that received accelerated approval for the treatment of patients with non-small cell lung cancer with *EGFR* exon 20 insertion mutations previously treated with platinum-based chemotherapy. This phase 1, 2-period, study was conducted to assess the absolute bioavailability of mobocertinib (Period 1), as well as mass balance, pharmacokinetics, metabolism, and excretion of [^14^C]-mobocertinib (Period 2) in healthy adult males. In Period 1, participants received a single oral capsule dose of 160 mg mobocertinib, followed by a 15-minute intravenous infusion of 50 µg (~ 2 µCi) [^14^C]-mobocertinib administered from 3.75 to 4 h after the capsule dose. In Period 2, a single oral dose of 160 mg (~ 100 µCi) [^14^C]-mobocertinib was administered as an oral solution. The geometric mean absolute bioavailability of mobocertinib was determined to be 36.7%. After oral administration of [^14^C]-mobocertinib, mobocertinib and its active metabolites, AP32960 and AP32914, were minor components in plasma, accounting for only 0.275% of total plasma radioactivity as the majority of mobocertinib-related material was covalently bound to plasma proteins. The geometric mean percentage of the administered radioactive dose recovered in the urine and feces was 3.57% and 76.0%, respectively. Only 0.39% of the oral dose of [^14^C]-mobocertinib was recovered in the urine as mobocertinib; thus, indicating that renal excretion of unchanged drug was a very minor pathway of elimination. In both treatment periods, mobocertinib was generally safe and well-tolerated as all adverse events were Grade 1 in severity. (*Trial registration number ClinicalTrials.gov NCT03811834. Registration date January 22, 2019).*

**Supplementary Information:**

The online version contains supplementary material available at 10.1007/s10637-024-01446-y.

## Introduction

Epidermal growth factor receptor (*EGFR)* exon 20 insertion (*EGFR*ex20ins) mutations represent 6–12% of all cases of *EGFR* mutated non-small cell lung cancer (NSCLC) [1–4]. Mobocertinib (TAK-788) is an oral tyrosine kinase inhibitor (TKI) that inhibits in-frame *EGFR*ex20ins mutations in NSCLC with better potency and selectivity over wild-type (WT) EGFR than other TKIs, including erlotinib, gefitinib, afatinib, and osimertinib [5]. Mobocertinib received accelerated approval in several countries for the treatment of adults with locally advanced or metastatic NSCLC with *EGFR*ex20ins mutations whose disease progressed on or after platinum-based chemotherapy [6, 7]. Accelerated approval was based on the results of a phase 1/2 study (EXCLAIM; NCT02716116) in 114 patients with platinum-pretreated *EGFR*ex20ins mutation positive locally advanced or metastatic NSCLC who received the recommended dose of mobocertinib (160 mg orally once daily [qd]). In this study, mobocertinib demonstrated durable responses, with an objective response rate per independent-review committee of 28%, median duration of response of 17.5 months, median progression-free survival of 7.3 months, and a median overall survival of 24.0 months [8]. The phase 3 EXCLAIM-2 study evaluated mobocertinib versus chemotherapy for the first-line treatment of *EGFR*ex20ins mutation positive locally advanced or metastatic NSCLC [9]. The primary endpoint of this phase 3 study was not met, thereby resulting in the initiation of a voluntary withdrawal of mobocertinib worldwide.

The pharmacokinetic (PK) profile of mobocertinib was characterized in healthy participants and in patients with NSCLC [10–12]. The median time to maximum observed concentration (t_max_) was 4 h after administration of oral doses ranging from 5 to 180 mg [10]. Following single- and multiple-dose administration, total combined molar systemic exposures of mobocertinib and its two active metabolites, AP32960 and AP32914, increased dose-proportionally over the 5 to 180 mg qd dose range [10, 11]. Both a low-fat and high-fat meal had no clinically meaningful effect on systemic exposures; therefore, mobocertinib may be administered with or without food [6, 11]. Mobocertinib is primarily metabolized by cytochrome P450 (CYP) 3A4/5 [5, 11] and the two active metabolites, AP32960 and AP32914, represent approximately 36% and 4% of the combined molar area under the plasma concentration–time curve (AUC), respectively [6]. The primary role of CYP3A-mediated metabolism to mobocertinib clearance was demonstrated in a drug-drug interaction study with the strong CYP3A inhibitor itraconazole and the strong CYP3A inducer rifampin, with itraconazole increasing the combined molar AUC of mobocertinib and its active metabolites by 527%, and rifampin decreasing the combined molar AUC by 95% [12]. In a population PK analysis using data from 427 subjects enrolled across 4 clinical studies, age, race, sex, body weight, mild-to-moderate renal impairment, or mild hepatic impairment had no clinically meaningful effect on mobocertinib PK; thus, no dose adjustment is recommended based on these covariates [13]. Exposure–response analyses showed that molar sum exposure to mobocertinib, AP32960, and AP32914 was not a statistically significant predictor of clinical response rates; however, time-averaged molar sum exposure was a significant predictor of the overall rate of grade ≥ 3 adverse events (AEs) [14].

The human absorption, distribution, metabolism, and excretion (ADME) study is an integral component of drug development that provides key information regarding the metabolic fate and excretion pathways for a drug [15]. Accordingly, this two-period phase 1 study was performed in healthy male adults to evaluate the absolute bioavailability of mobocertinib in Period 1, as well as the mass balance, PK, metabolism, and routes of excretion of [^14^C]-mobocertinib after administration of a single oral dose in Period 2.

## Methods

### Study design

This was a two-period, open-label, single-dose phase 1 study (NCT03811834) conducted at one clinical site (Celerion, Lincoln, Nebraska, USA) in healthy male participants. The primary objective of Period 1 was to determine the absolute bioavailability of mobocertinib following a single oral dose of 160 mg mobocertinib as capsules and a single intravenous (IV) microdose of 50 µg (~ 2 µCi) [^14^C]-mobocertinib. The primary objectives of Period 2 were to (1) assess the cumulative excretion of total radioactivity in urine and feces (mass balance); (2) assess the metabolite profile of mobocertinib in plasma, urine, and feces; and (3) characterize the PK of mobocertinib, AP32960, and AP32914 in plasma, whole blood, and urine, and total radioactivity concentration equivalents in plasma and whole blood following a single oral dose of 160 mg (~ 100 µCi) [^14^C]-mobocertinib as an oral solution. A sample size of 6 healthy males was selected without statistical considerations and deemed adequate to meet the study objectives. Furthermore, the sample size was limited based on clinical considerations for human ADME studies and to limit exposure to radioactivity.

### Period 1: Absolute Bioavailability Study

On Day 1 of Period 1, following an overnight fast of at least 10 h, participants received a single 160 mg dose of non-radiolabeled mobocertinib as capsules. At 3.75 h post oral dosing (i.e., 15 min before the median oral t_max_ of ~ 4 h), participants received a nominal dose of 50 µg (~ 2 µCi) [^14^C]-mobocertinib as a 15-minute IV infusion. However, due to nonspecific binding of [^14^C]-mobocertinib to the dosing syringe and tubing, actual doses administered ranged from 36.8 to 38.7 µg (1.47 to 1.55 µCi). Participants were required to stay in the clinic from Day − 1 through at least the 96-hour blood draw (Day 5) or until a discharge criterion was met (i.e., ≥ 80% of the total administered radioactive dose was recovered in urine and fecal samples or excretion of radioactivity in urine and feces combined had declined to ≤ 1% of the total administered dose per day for ≥ 2 consecutive intervals), up to a maximum of 7 days postdose (Day 8). Blood samples for the measurement of plasma mobocertinib, AP32960, and AP32914 concentrations were collected predose and at 0.5, 1, 2, 3, 4, 5, 6, 8, 12, 24, 36, 48, 72, and 96 h after oral administration. Additional blood samples to determine [^14^C]-total radioactivity, [^14^C]-mobocertinib, [^14^C]-AP32960, and [^14^C]-AP32914 in plasma were collected at 3.75 h after oral dosing (i.e., predose for [^14^C] assessments), at the end of the IV infusion, and at 10, 20, and 30 min, and 1, 2, 4, 8, 20, 32, 44, 68, and 92 h after the end of infusion. For participants who did not meet the discharge criteria by Day 5, blood samples continued to be collected in 24-hour intervals until a discharge criterion was met or up to Day 8. Urine and feces were collected before oral dosing (− 48–0 h); urine was collected over 0–3.75 h, 3.75–12 h and 12–24 h after oral dosing; feces were collected from 0 to 3.75 h and 3.75–24 h after oral dosing; both urine and feces were then collected over 24-hour intervals until a discharge criterion was met, or up to Day 8 (168 h postdose).

### Period 2: Human ADME Study 

After a washout of 8–9 days, participants returned to the clinic for Day − 1 of Period 2. On Day 1 of Period 2, after a fast of ≥ 10 h, participants received a single nominal dose of 160 mg (~ 100 µCi; whole body effective dose of 5.3 mrem for a 70-kg male human) [^14^C]-mobocertinib as a 70 mL oral solution. The actual doses administered ranged from 161 to 163 mg (92.4 to 93.9 µCi). Participants stayed in the clinic from Day − 1 until a discharge criterion (≥ 80% of the total administered radioactive dose was recovered in urine and feces or excretion of radioactivity in urine and feces combined had declined to ≤ 1% of the total administered dose per day for ≥ 2 consecutive intervals) was met, or up to 10 days postdose. To measure the total radioactivity and concentrations of mobocertinib, AP32960, and AP32914 in whole blood and plasma, 4 blood samples were collected predose, and at 0.5, 1, 2, 3, 4, 5, 6, 8, 12, 24, 36, 48, 72, 96, 120, 144, 168, 192, 216, and 240 h postdose; a fifth blood sample was collected for plasma metabolite profiling at predose and 1, 2, 4, 6, 12, 24, 48, 72, 96, 120, and 168 h postdose. Feces and urine were collected predose (feces within 48 h and urine within 24 h prior to dosing), 0–24 h (urine from 0–12 and 12–24 h), and then at 24-hour intervals up to 240 h postdose or until a discharge criterion was met. Two participants did not meet a discharge criterion by Day 11. These 2 participants continued with at-home fecal sample collections. One participant provided fecal samples until Day 13 (264–288 h interval) and one participant provided fecal samples until Day 19 (408–432 h interval), after which they met a discharge criterion.

All participants were contacted 30 days after the last dose of study drug for safety follow-up.

### Participants

Eligible participants were healthy, adult, male non-smokers, aged 19–55 years with a body mass index ≥18.0 and < 30.0 kg/m^2^ and medically healthy with no clinically significant medical history, physical examination, laboratory profile, vital sign, or electrocardiogram (ECG) findings. Key exclusion criteria were QT interval corrected for heart rate using Fridericia’s formula (QTcF) interval > 460 ms; estimated creatinine clearance < 80 mL/min; infrequent bowel movements within the previous 30 days or recent history of abnormal bowel movements (e.g., diarrhea, constipation) within 2 weeks before first dose; inability to refrain from use of any prescription or non-prescription medication, herbal remedy, or vitamin supplement within 2 weeks before first dose; and use of inducers of CYP3A and/or P-glycoprotein within 28 days before first dose and throughout the study. Full eligibility criteria are provided in Supplementary Table S1.

### Bioanalytical methods

Plasma, whole blood, and urine samples were assayed for mobocertinib, AP32960, and AP32914 concentrations using liquid chromatography-tandem mass spectrometry (Q2 Solutions, Ithaca, New York, USA). The analytical range for each analyte was 0.250 to 500 ng/mL in whole blood and plasma, and 1.00 to 1000 ng/mL in urine. Plasma, urine, and fecal samples from Period 1 were assayed for [^14^C]-mobocertinib, [^14^C]-AP32960, and [^14^C]-AP32914 concentrations, and plasma samples were assayed for total radioactivity concentration equivalents using accelerator mass spectrometry (AMS; Pharmaron, Germantown, Maryland, USA), with lower limits of quantitation (LLOQs) for each analyte of 1.20 pg/mL in plasma and 6.99 pg/mL in urine and feces; the LLOQ for total radioactivity concentration equivalents in plasma ranged from 0.972 to 2.00 pg eq/mL across participants. Urine and fecal samples were assayed for total radioactivity following the IV dose of [^14^C]-mobocertinib in Period 1, and whole blood, plasma, urine, and fecal samples were assayed for total radioactivity following the oral dose of [^14^C]-mobocertinib in Period 2 using liquid scintillation counting (LSC; Celerion, Lincoln, Nebraska, USA; LLOQs: Period 1 urine: 0.115–0.118 ng eq/g, feces: 0.720–0.759 ng eq/g; Period 2 whole blood: 87.3–112 ng eq/g, plasma: 116–132 ng eq/mL, urine: 37.8–46.8 ng eq/g, feces: 110–336 ng eq/g across participants). Urine and fecal samples collected in Period 1 that had total radioactivity concentration equivalents below the LLOQ of the Celerion LSC assay were sent to Pharmaron for measurement by AMS (LLOQ: urine: 0.0000253–0.00111 ng eq/g; feces: 0.000404–0.0278 ng eq/g across participants). The LSC and AMS assays were not cross-validated.

### Pharmacokinetic analyses

Noncompartmental PK parameters were calculated for analytes based on total concentrations in whole blood and plasma using Phoenix WinNonlin versions 7.0 and 8.1 (Certara, Princeton, New Jersey, USA). Derived PK parameters included t_max_, maximum observed concentration (C_max_), AUC from time 0 to the last quantifiable concentration (AUC_last_), AUC from time 0 to infinity (AUC_∞_), apparent clearance after oral administration (CL/F; mobocertinib only), and apparent volume of distribution during the terminal disposition phase after oral administration (V_z_/F; mobocertinib only). In Period 1, mobocertinib clearance (CL) and volume of distribution during the terminal disposition phase (V_z_) were calculated after IV administration. Blood-to-plasma ratios for C_max_ and AUC_∞_ were calculated for mobocertinib, AP32960, and AP32914 in Period 2. The absolute bioavailability of mobocertinib was estimated by comparing the ln-transformed AUC_∞_ of mobocertinib in plasma following the single oral dose of mobocertinib 160 mg as capsules and the ln-transformed dose-normalized (to 160 mg) AUC_∞_ of [^14^C]-mobocertinib following the single IV microdose in Period 1 using an analysis of variance model with route of administration (oral/IV) as a fixed effect and participant as a random effect. The geometric mean ratio and 90% CIs were expressed as a percentage relative to IV administration.

PK parameters for analytes in urine and feces were calculated using SAS Version 9.4 and included the cumulative amounts and percentages of the administered radioactive dose excreted as [^14^C]-mobocertinib, [^14^C]-AP32960, and [^14^C]-AP32914 in urine and feces following IV dosing in Period 1 and oral dosing in Period 2. Renal clearance (CL_R_) was calculated as the cumulative amount of radiolabeled mobocertinib, AP32960, or AP32914 recovered in urine divided by the plasma AUC to the time of the last common time point at which an analyte was quantifiable in both urine and plasma for individual participants (AUCCL_R_) following oral dosing of [^14^C]-mobocertinib in Period 2. Mass balance was defined as the percentage of total radioactivity recovered in urine and feces combined relative to the total amount of the administered radioactive dose.

### Safety assessments

Safety was evaluated based on the incidence and severity of AEs and changes from baseline in clinical laboratory results, vital signs, and ECG parameters. AEs were graded according to Common Terminology Criteria for Adverse Events version 5.0.

## Results

### Participants

Seven healthy male participants enrolled in the study and received treatment; 6 of whom completed the study. One participant discontinued on Day 2 of Period 1 because of multiple AEs and was replaced. PK analyses were performed on data from the 6 participants who completed the study. Most participants were white (5/7; 71%). The median age was 33.0 years (range, 23–55 years) and the median BMI was 27.45 kg/m^2^ (range, 24.64–29.37 kg/m^2^).

### Period 1: Absolute Bioavailability Study

#### Oral and IV PK in plasma

**Fig. 1:**
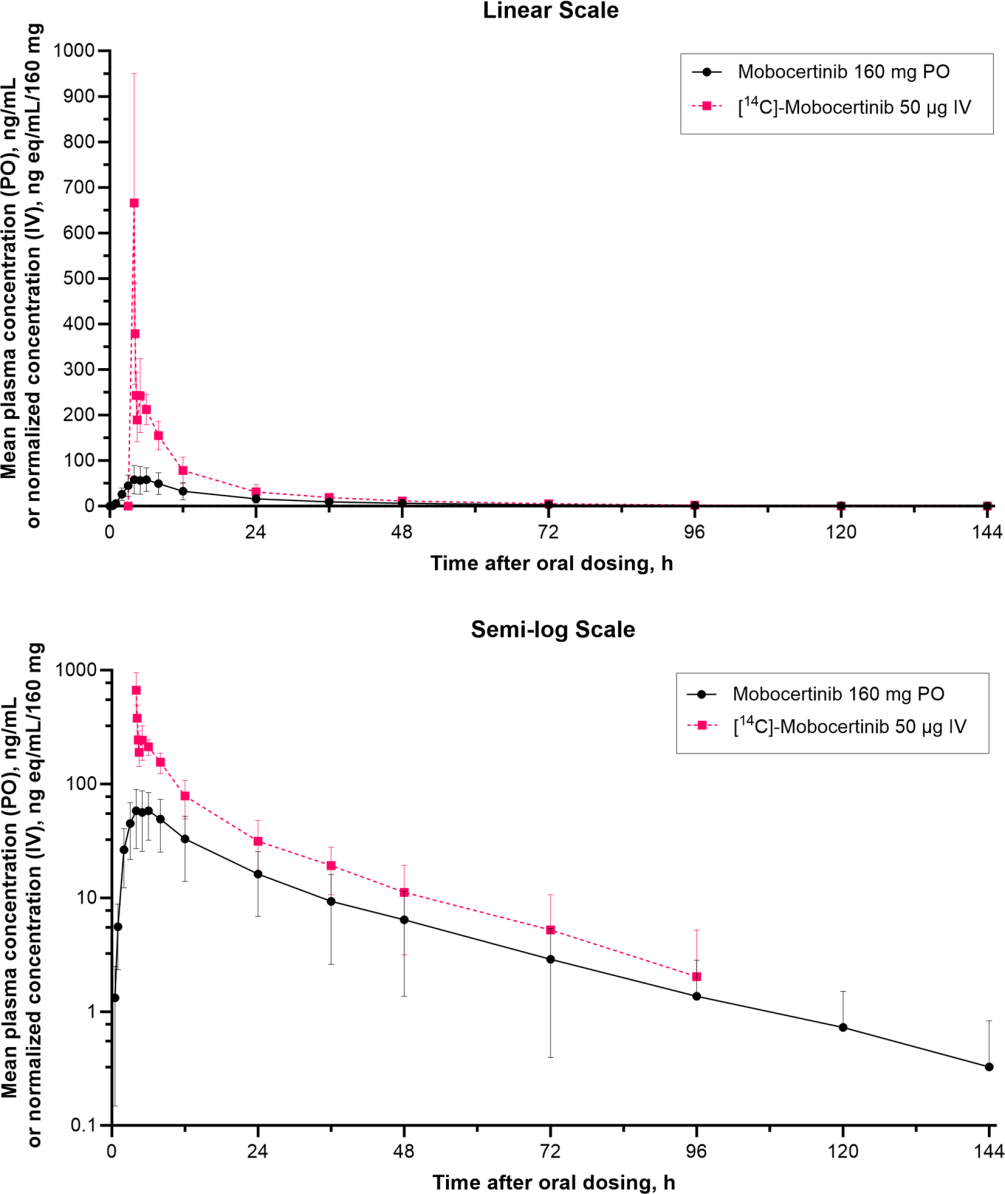
Arithmetic mean (standard deviation) plasma concentration-time profiles of mobocertinib and [^14^C]-mobocertinib (normalized to a 160 mg dose for the IV profile only) following administration of a single oral dose of 160 mg mobocertinib and a single IV dose of 50 µg (~ 2 µCi) [^14^C]-mobocertinib administered over 15 min from 3.75 to 4 h after the oral dose in Period 1. *IV* intravenous, *PO* oral

Arithmetic mean plasma concentrations of mobocertinib and [^14^C]-mobocertinib (normalized to a 160 mg dose) following a single 160 mg oral dose and IV infusion of 50 µg (~ 2 µCi) [^14^C]-mobocertinib co-administered over 15 min from 3.75 to 4 h after oral dosing are shown in Fig. 1. Plasma concentrations of [^14^C]-mobocertinib normalized to a 160 mg dose following IV co-administration were higher than concentrations following oral dosing throughout the entire sampling interval, with both analytes exhibiting similar elimination profiles. PK parameters are summarized in Table 1. Following oral administration, the median t_max_ was similar for mobocertinib and its active metabolites, ranging from 4.5 to 5.0 h. After IV administration, the median t_max_ for metabolites [^14^C]-AP32960 (3.3 h) and [^14^C]-AP32914 (2.3 h) occurred ~ 2 to 3 h after [^14^C]-mobocertinib (0.26 h). The geometric mean AUC_∞_ value for mobocertinib following the 160 mg oral dose was 36.7% of the dose-normalized AUC_∞_ value after IV dosing. Therefore, the geometric mean absolute oral bioavailability for mobocertinib was moderate at 36.7% (90% CI, 22.4–60.2%).

**Table 1:** Summary of plasma PK parameters following single-dose oral and IV administration of mobocertinib in Period 1

	Oral mobocertinib 160 mg			IV infusion [^14^C]-mobocertinib 50 µg (~ 2 µCi) ^a^		
Parameter	Mobocertinib *n* = 6	AP32960 *n* = 6	AP32914 *n* = 6	[^14^C]-mobocertinib *n* = 6	[^14^C]-AP32960 *n* = 6	[^14^C]-AP32914 *n* = 2^b^
t_max,_ h^c^	5.0 (4.0–6.0)	4.5 (4.0–6.0)	5.0 (4.0–6.0)	0.26 (0.26–1.25)	3.3 (2.3–8.3)	2.3 (2.3–2.3)
C_max,_ ng/mL^d, e^	56.7 (52.2)	23.3 (21.2)	4.1 (36.0)	624.9 (43.6)	27.9 (78.6)	16.2 (35.5)
AUC_∞_, ng*h/mL^d, e^	1050 (64.9)	478 (30.6)	73.0 (53.2)	2864 (30.0)	793 (61.5)^f^	390^g^
t_1/2z_, h^d^	22.9 (17.9)	29.1 (18.2)	12.5 (37.5)	22.2 (41.3)	20.3 (39.1)^f^	11.9^g^
CL/F, L/h^d, h^	152 (64.9)	NA	NA	55.9 (30.0)	NA	NA
V_z_/F, L^d, h^	5030 (53.6)	NA	NA	1791 (45.3)	NA	NA
Molar AUC_∞_ M:P ratio^d^	NA	0.466 (33.9)	0.071 (23.1)	NA	0.300 (46.4)^f^	0.128^g^

*AUC*_∞_ area under the concentration-time curve from time 0 to infinity, *C*_*max*_ maximum observed concentration, *CL/F* apparent clearance after oral administration, *CV%* percentage coefficient of variation, *IV* intravenous, *M:P* metabolite-to-parent, *NA* not applicable, *PK* pharmacokinetic, *t*_*1/2z*_ terminal disposition phase half-life, *t*_*max*_ time of first occurrence of C_max_, *V*_*z*_*/F* apparent volume of distribution during the terminal disposition phase after oral administration

^a^ Single IV infusion of 50 µg (~ 2 µCi) [^14^C]-mobocertinib administered over 15 min from 3.75 to 4 h after administration of the single oral dose of 160 mg mobocertinib

^b^ PK parameters for [^14^C]-AP32914 were not calculated for 4 participants because they had fewer than 3 consecutive measurable plasma [^14^C]-AP32914 concentrations

^c^ Median (range)

^d^ Geometric mean (geometric CV%)

^e^ To allow comparisons to oral dosing values, exposure parameters C_max_ and AUC_∞_ estimated after IV tracer administration were normalized to a 160 mg dose

^f^*n* = 5

^g^*n* = 1

^h^ After IV administration, values represent CL and V_z_

#### Excretion in urine and feces

Following an IV infusion of 50 µg (~ 2 µCi) [^14^C]-mobocertinib, 1.5%, 0.6%, and 0.0% of the administered radioactive dose was excreted in urine as [^14^C]-mobocertinib, [^14^C]-AP32960, and [^14^C]-AP32914, respectively, which was less than the 4.7% of the dose recovered as total radioactivity in urine overall. In feces, 2.5%, 7.1%, and 0.3% of the radioactive dose was excreted as [^14^C]-mobocertinib, [^14^C]-AP32960, and [^14^C]-AP32914, respectively, which was much lower than the 69.9% of the dose recovered as total radioactivity in feces overall.

### Period 2: Human ADME Study

#### Plasma and whole blood PK

**Fig. 2:**
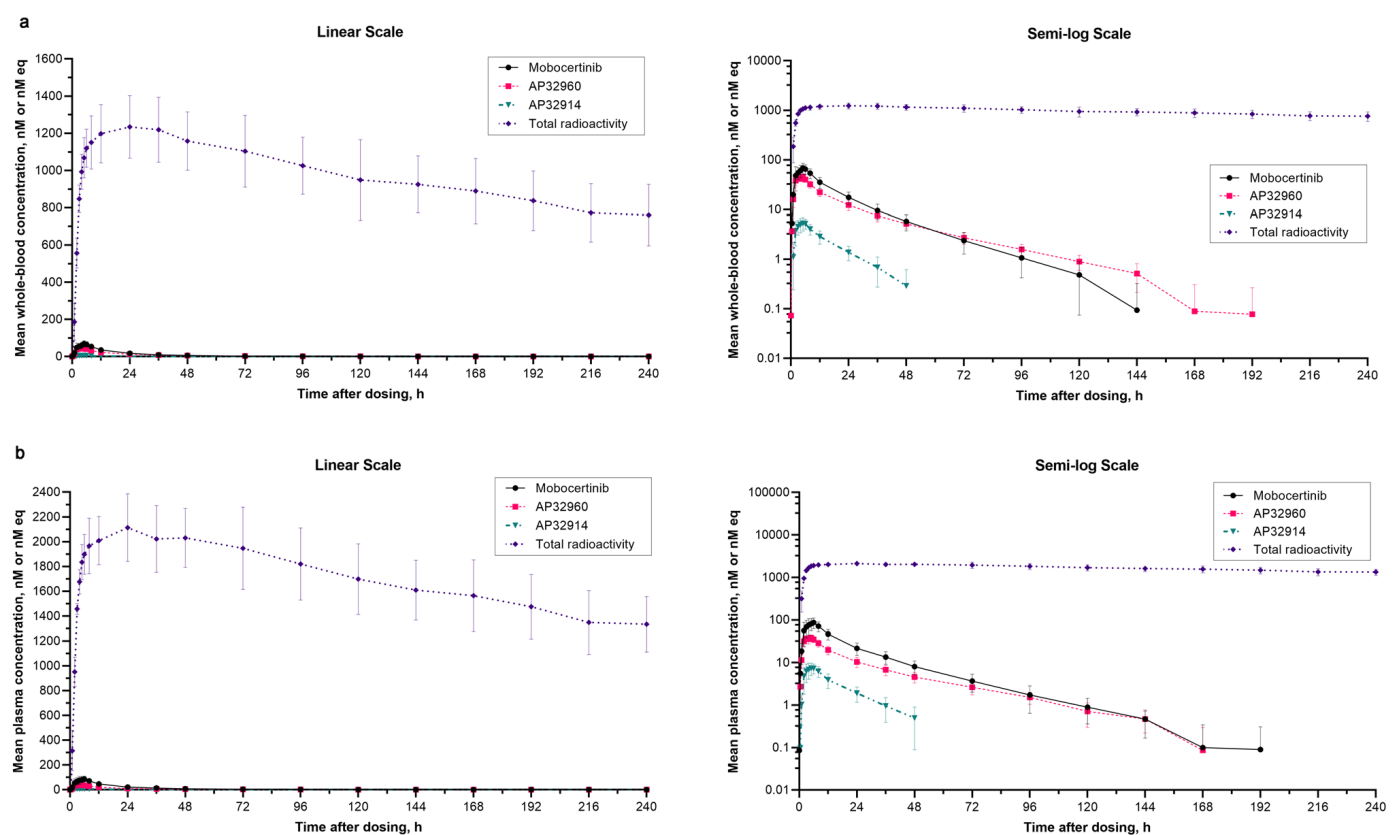
Arithmetic mean (standard deviation) (**a**) whole blood and (**b**) plasma molar concentrations of mobocertinib, AP32960, AP32914 and total radioactivity (in molar concentration equivalents) versus timeprofiles following administration of a single oral solution dose of 160 mg (~ 100 µCi) [^14^C]-mobocertinib in Period 2

The arithmetic mean molar concentration versus time profiles for mobocertinib, AP32960 and AP32914, as well as the total radioactivity molar concentration equivalents versus time profile, following a single oral solution dose of 160 mg (~ 100 µCi) [^14^C]-mobocertinib are shown for whole blood in Fig. 2a and for plasma in Fig. 2b. The concentration-time profiles for mobocertinib, AP32960, AP32914, and total radioactivity were generally similar in appearance between whole blood and plasma. However, arithmetic mean concentrations of mobocertinib, AP32960, and AP32914 were much lower than those for total radioactivity in whole blood and plasma. The arithmetic mean blood-to-plasma (B:P) concentration ratios for mobocertinib, AP32960, AP32914, and total radioactivity ranged from 0.60 to 1.04, 1.03 to 1.50, 0.66 to 1.04, and 0.56 to 0.60, respectively.

PK parameters for mobocertinib, AP32960, AP32914, and total radioactivity in whole blood and plasma are summarized in Table 2. Median t_max_ values were similar between whole blood and plasma for mobocertinib, AP32960, AP32914, and total radioactivity. The geometric mean C_max_ and AUC_∞_ values for mobocertinib and AP32914 were slightly lower in whole blood than in plasma, whereas the AP32960 C_max_ and AUC_∞_ values were slightly higher in whole blood than in plasma. Specifically, the geometric mean AUC_∞_ values of mobocertinib, AP32960, and AP32914 were 24% lower, 15% higher, and 29% lower, respectively, in whole blood compared to plasma. Based on the molar AUC_∞_ ratios, mobocertinib, AP32960, and AP32914 (combined) accounted for 0.275% of total plasma radioactivity. Geometric mean t_1/2z_ values of mobocertinib, AP32960, AP32914, and total radioactivity in whole blood were comparable to those observed in plasma.

**Table 2:** Summary of whole blood and plasma PK parameters following a single oral solution dose of 160 mg (~ 100 µCi) [^14^C]-mobocertinib in Period 2

Parameter	Whole blood				Plasma			
	Mobocertinib *n* = 6	AP32960 *n* = 6	AP32914 *n* = 6	Total radioactivity *n* = 6	Mobocertinib *n* = 6	AP32960 *n* = 6	AP32914 *n* = 6	Total radioactivity *n* = 6
t_max,_ h^a^	5.0 (2.0–6.0)	5.0 (3.0–5.0)	5.0 (4.0–6.0)	24.0 (8.0–36.0)	6.0 (3.0–6.0)	4.0 (3.0–5.0)	6.0 (3.0–6.0)	24.0 (8.0–36.0)
C_max,_ ng/mL^b, c^	40.5 (29.1)	26.7 (10.6)	3.05 (28.6)	725 (14.6)	51.0 (34.9)	22.7 (19.8)	4.28 (34.7)	1250 (13.4)
AUC_last,_ ng*h/mL^b, c^	718 (33.2)	543 (20.5)	45.7 (40.4)	131,000 (18.5)	945 (39.2)	471 (27.5)	63.6 (45.7)	230,000 (15.5)
AUC_∞_, ng*h/mL^b, c^	729 (32.8)	556 (20.2)	52.4 (37.3)	325,000 (21.2)	956 (38.6)	486 (26.7)	73.4 (39.9)	556,000 (14.9)
t_1/2z,_ h^b^	20.5 (25.4)	30.9 (12.1)	12.8 (23.1)	301 (28.1)^d^	22.8 (26.6)	30.5 (21.1)	14.0 (30.6)	281 (29.1)^d^
CL/F, L/h^b^	222 (32.6)	NA	NA	0.498 (20.7)	169 (38.4)	NA	NA	0.292 (14.6)
V_z_/F, L^b^	6550 (18.0)	NA	NA	216 (23.7)	5560 (16.6)	NA	NA	118 (22.8)
Molar AUC_∞_ M:P ratio^b^	NA	0.781 (12.3)	0.0736 (15.7)	NA	NA	0.520 (15.3)	0.0787 (15.1)	NA
Molar AUC_∞_ ratio (analyte vs. total radioactivity)^b^	0.00224 (28.3)	0.00175 (21.6)	0.000165 (31.2)	NA	0.00172 (31.1)	0.000896 (21.4)	0.000135 (36.1)	NA
B:P Ratio C_max_^b^	0.794 (17.1)	1.18 (10.4)	0.713 (8.10)	NA	NA	NA	NA	NA
B:P Ratio AUC_∞_^b^	0.763 (17.7)	1.15 (10.2)	0.714 (7.60)	NA	NA	NA	NA	NA

*AUC*_∞_ area under the concentration-time curve from time 0 to infinity, *AUC*_*last*_ area under the concentration-time curve from time 0 to the last quantifiable concentration, *B:P* blood-to-plasma, *C*_*max*_ maximum observed concentration, *CL/F* apparent clearance after oral administration, *CV%* percentage coefficient of variation, *M:P* metabolite-to-parent, *NA* not applicable, *PK* pharmacokinetic, *t*_*1/2z*_ terminal disposition phase half-life, *t*_*max*_ time of first occurrence of C_max_, *V*_*z*_*/F* apparent volume of distribution during the terminal disposition phase after oral administration

^a^ Median (range)

^b^ Geometric mean (geometric CV%)

^c^ Mass units of parameter values for mobocertinib, AP32960, and AP32914 are presented in ng. Mass units of parameter values for total radioactivity are presented in ng equivalents

^d^ The t_1/2z_ values for total radioactivity exceed the duration of sampling, thereby making them approximate estimates

#### Urine PK

The geometric mean (percentage coefficient of variation [CV]) renal clearances of mobocertinib, AP32960, and AP32914 were 0.657 L/h (44.3%), 1.95 L/h (22.5%), and 2.85 L/h (27.9%), respectively. The geometric mean (CV) percentage of the total oral radioactive dose recovered in urine as [^14^C]-mobocertinib, [^14^C]-AP32960, and [^14^C]-AP32914 was 0.39% (31.3%), 0.60% (16.3%), and 0.14% (23.1%), respectively; therefore, 1.13% of the radioactive dose was recovered as all three analytes combined.

#### Mass balance

**Fig. 3:**
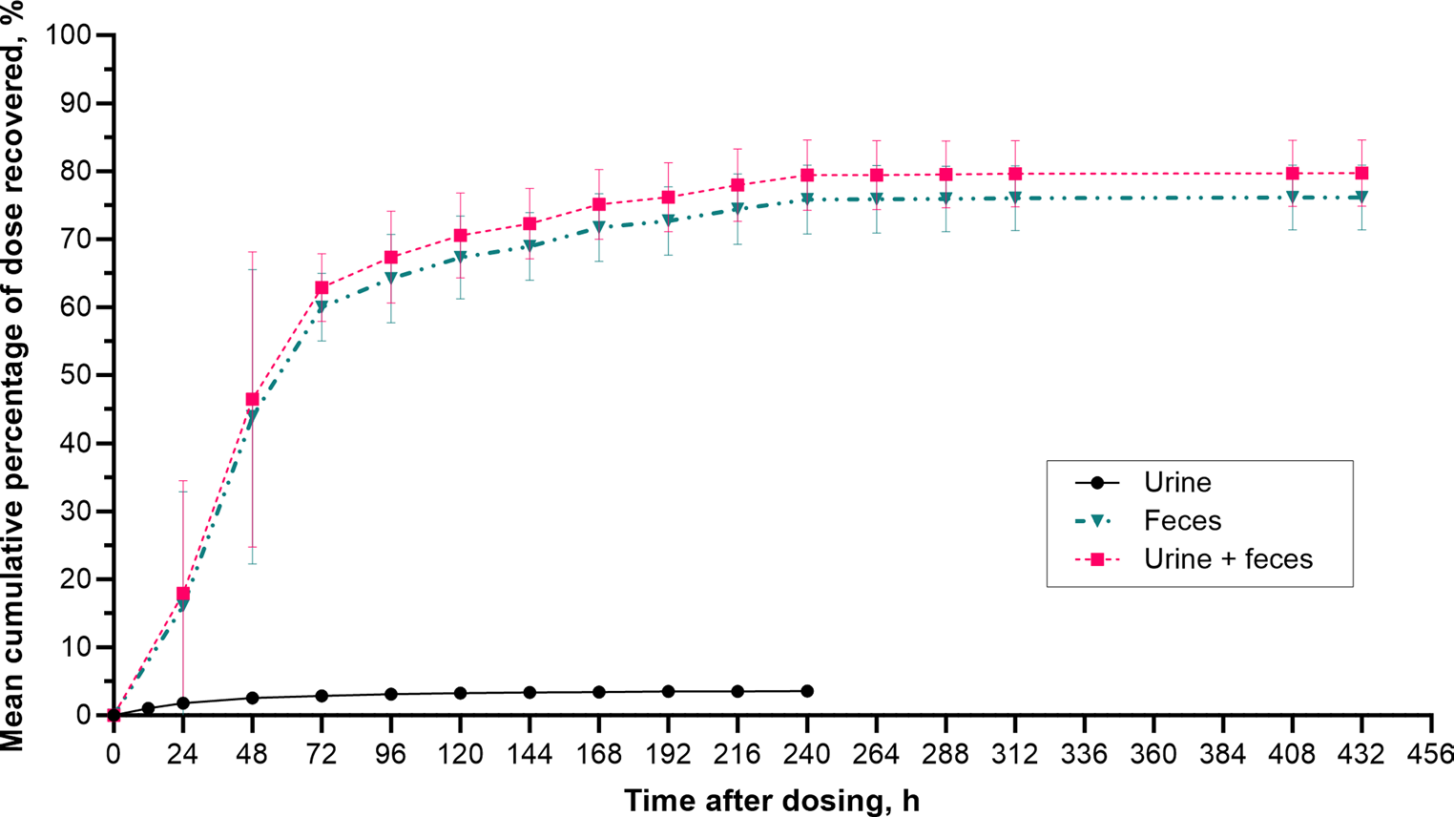
Arithmetic mean (standard deviation) cumulative percentage of dose recovered as total radioactivity in urine, feces, and combined urine and feces versus time profiles following administration of a single oral solution dose of 160 mg (~ 100 µCi) [^14^C]-mobocertinib in Period 2

The arithmetic mean cumulative percentages of the radioactive dose recovered in urine, feces, and combined (urine and feces) over time following oral administration of [^14^C]-mobocertinib are shown in Fig. 3. The geometric mean cumulative percentage of the radioactive dose recovered was 3.57% (range, 3.12–4.44%) in urine, 76.0% (range, 67.0–79.8%) in feces, and 79.6% (range, 70.2–83.2%) in both excreta combined.

### Safety

There were no deaths or serious AEs. All 7 participants experienced at least 1 AE, including 6 participants in Period 1 and 4 in Period 2. The most common AEs were constipation, diarrhea, and nausea, each reported in 2 of 7 participants (29%). All AEs were grade 1 (mild) in severity. One participant discontinued the study early because of multiple AEs (eructation, nausea, salivary hypersecretion, and vomiting on Day 1 of Period 1).

## Discussion

In this phase 1 study, the absolute bioavailability of mobocertinib was determined using an IV microtracer dosing approach (Period 1), followed by the characterization of the human ADME properties of mobocertinib and its active metabolites after oral administration (Period 2). The approach of concomitantly dosing with a radiolabeled IV microdose after administration of a non-labelled therapeutic oral dose to determine absolute bioavailability has been adequately established and offers advantages over traditional crossover absolute bioavailability studies [16]. In addition, administration of the IV microtracer dose of mobocertinib in Period 1 enabled the determination of key pharmacokinetic parameters, including clearance and volume of distribution, which cannot be characterized as part of a standard ADME study involving only oral administration [15].

The absolute bioavailability of mobocertinib was moderate at 36.7%. CYP3A is the primary enzyme involved in the metabolism of mobocertinib [6, 17]. In a drug-drug interaction study in healthy participants, coadministration of the strong CYP3A inhibitor itraconazole significantly increased the combined molar AUC_∞_ of mobocertinib and its active metabolites by 527%, whereas coadministration of the strong CYP3A inducer rifampin significantly decreased the combined molar AUC_∞_ by 95%; thereby, demonstrating the predominant role of CYP3A-mediated metabolism to mobocertinib disposition [12]. CYP3A is abundantly expressed in the liver and gastrointestinal tract, and accounts for approximately 80% of total CYP450 in the intestine [18, 19]. Extensive metabolism by CYP3A in the gastrointestinal tract and/or liver can lead to poor-to-moderate oral bioavailability for CYP3A substrates [18]. Thus, the moderate oral bioavailability of mobocertinib is likely explained by first-pass metabolism via CYP3A.

Following oral administration of [^14^C]-mobocertinib in Period 2 (human ADME study), mobocertinib and its active metabolites, AP32960 and AP32914, were minor components in plasma (0.275% of total plasma radioactivity). The majority of mobocertinib-related radioactivity in plasma was likely covalently bound to plasma proteins as mobocertinib is capable of forming covalent bonds with other proteins (i.e., WT EGFR and *EGFR*ex20ins-mutated EGFR) [5]. Mobocertinib and its active metabolites did not show preferential distribution into human whole blood over plasma as the B:P ratios for C_max_ and AUC_∞_ were generally close to 1.

Adequate recovery (79.6%) of orally administered mobocertinib-related radioactivity was achieved over the 432-hour collection interval after administration of the 160 mg radiolabeled dose, with predominant excretion in feces (76.0%) and only a small amount excreted in urine (3.57%). Approximately 20% of the administered radioactive dose was unaccounted for, likely due to the slow excretion of covalently bound drug-related material. The total recovery is also consistent with the long approximate half-life estimate of 281 h observed for plasma total radioactivity. Of note, a similar total recovery of 82% has been reported from the human ADME study for osimertinib, another irreversible EGFR TKI [20].

Consistent with the limited excretion of total radioactivity in the urine, renal clearance of mobocertinib was low and only 1.13% of the dose was recovered in urine as mobocertinib, AP32960, and AP32914 combined. Collectively, these findings indicate that renal excretion is not a major pathway of elimination for mobocertinib and align with the results of the population PK analysis that demonstrated no clinically meaningful effect of mild-to-moderate renal impairment on mobocertinib PK [13]. Furthermore, additional metabolites appear to be excreted in the urine as the percentage of the administered dose recovered in urine that was attributable to mobocertinib, AP32960, and AP32914 combined (1.13%) was lower than the overall percentage of total radioactivity recovered in the urine (3.57%). The results of additional metabolite profiling analyses using the plasma, urine, and fecal samples collected in this study will be reported separately.

The safety profile of mobocertinib was consistent with previous clinical experience [8, 10], with no new safety signals observed. All AEs were Grade 1 (mild) in severity.

In conclusion, this study demonstrated that mobocertinib has moderate oral absolute bioavailability and fecal excretion represents the primary route of excretion of mobocertinib-related material.

### Electronic supplementary material

Below is the link to the electronic supplementary material.

Supplementary Material 1

## Data Availability

Takeda does not plan to share data supporting the results reported in this article as there is a reasonable likelihood that study participants could be re-identified.
