# Supplementary material for: A phase 1 study to assess the absolute bioavailability, mass balance, pharmacokinetics, metabolism, and excretion of [14C]-mobocertinib, an oral inhibitor of EGFR exon 20 insertion mutations, in healthy participants
Source: Invest New Drugs. 2024 May 24;42(4):343–52. doi: 10.1007/s10637-024-01446-y (PMC11327196; doi:10.1007/s10637-024-01446-y)
Supplement: Supplementary file 1 — Supplementary Material 1 [file 10637_2024_1446_MOESM1_ESM.pdf]

Title: A phase 1 study to assess the absolute bioavailability, mass balance, pharmacokinetics, and excretion of [^{14}C]-mobocertinib, an oral EGFR inhibitor, in healthy participants

Jounal: Investigational New Drugs

Authors: Michael J. Hanley[a]; Steven Zhang[b]; Robert Griffin[c]; Sean Xiaochun Zhu[d]; Robert J. Frame[e]; Jianchang Lin[f]; Karthik Venkatakrishnan[g]; Neeraj Gupta[h]

[a]Quantitative Clinical Pharmacology, Takeda Development Center Americas, Inc., Lexington, Massachusetts, USA; ORCID ID: 0000-0001-9266-2797; [b]Quantitative Clinical Pharmacology, Takeda Development Center Americas, Inc., Lexington, Massachusetts, USA; [c]Global DMPK, Takeda Development Center Americas, Inc., Lexington, Massachusetts, USA; ORCID ID: 0000-0002-1907-2421; [d]Global DMPK, Takeda Development Center Americas, Inc., Lexington, Massachusetts, USA; ORCID ID: 0009-0006-7937-812X; [e]Clinical Science, Takeda Development Center Americas, Inc., Lexington, Massachusetts, USA; ORCID ID: 0000-0001-7216-3130; [f]Statistical & Quantitative Sciences, Takeda Development Center Americas, Inc., Lexington, Massachusetts, USA; [g]Quantitative Pharmacology, EMD Serono Research & Development Institute, Inc., Billerica, Massachusetts, USA; ORCID 0000-0003-4039-9813; This author was an employee of Millennium Pharmaceuticals, Inc., a wholly owned subsidiary of Takeda Pharmaceutical Company Limited, Cambridge, Massachusetts, USA, at the time the study was conducted; [h]Quantitative Clinical Pharmacology, Takeda Development Center Americas, Inc., Lexington, Massachusetts, USA; ORCID ID: 0000-0002-5500-5218.

Address for correspondence:

Primary Author's name:	Michael J. Hanley
Title:	Director
Affiliation:	Quantitative Clinical Pharmacology, Takeda Development Center Americas, Inc
Address	95 Hayden Avenue, Lexington, MA, 02421 USA
Phone:	617-444-1346
E-mail:	Michael.Hanley@takeda.com

SUPPLEMENTARY MATERIALS

Table S1 Inclusion and exclusion criteria

**Inclusion criteria**
Participants fulfilled the following inclusion criteria to be eligible for participation in the study:<br>1. Healthy adult male, 19–55 years of age, inclusive, at screening.<br>2. Continuous non-smoker who had not used nicotine-containing products for ≥20 years prior to the first dose and throughout the study, based on participant self-report.<br>3. Body mass index (BMI) ≥18 and <30.0 kg/m^2 at screening.<br>4. Medically healthy with no clinically significant medical history, physical examination, laboratory profiles, vital signs, or ECGs, as deemed by the investigator or designee.<br>5. Normal baseline pulmonary function tests (≥80% of predicted normal for spirometry and lung volumes) within 7 days prior to the first dose.<br>6. Participants who were sexually active with a female partner of childbearing potential must have used barrier contraception or abstain from sexual intercourse during the study until 94 days after the last dose. Total abstinence (no sexual intercourse) was considered an acceptable method of birth control if it agreed with the participant's preferred and usual lifestyle.<br>7. Must have agreed not to donate sperm from the first dose until 94 days after the last dose.<br>8. Understood the study procedures in the informed consent form and was willing and able to comply with the protocol.
**Exclusion criteria**
A participant was excluded from the study if the participant:<br>1. Was mentally or legally incapacitated or had significant emotional problems at the time of the screening visit or expected during the conduct of the study.<br>2. History or presence of clinically significant medical or psychiatric condition or disease in the opinion of the investigator or designee.<br>3. History of any illness that, in the opinion of the investigator or designee, might have confounded the results of the study or posed an additional risk to the participant by their participation in the study.<br>4. History or presence of alcoholism or drug abuse within the past 2 years prior to the first dose.<br>5. History or presence of hypersensitivity or idiosyncratic reaction to the study drug or related compounds.<br>6. History or presence of lung disease and current lung infection.<br>7. Positive urine drug or alcohol results at screening or first check-in.<br>8. Positive results at screening for human immunodeficiency virus (HIV), hepatitis B surface antigen (HBsAg), or hepatitis C virus (HCV).<br>9. Seated blood pressure was less than 90/40 mmHg or greater than 140/90 mmHg at screening.<br>10. Seated heart rate was lower than 40 bpm or higher than 99 bpm at screening.<br>11. QT interval corrected for heart rate using Fridericia's formula (QTcF) interval was >460 ms or ECG findings were deemed abnormal with clinical significance by the investigator or designee at screening.<br>12. Estimated creatinine clearance <80 mL/min at screening.<br>13. Had tattoo(s) or scarring at or near the site of IV infusion or any other condition which may have interfered with infusion site examination, in the opinion of the investigator.<br>14. Participant had infrequent bowel movements (less than approximately once per day) within 30 days prior to first dose.<br>15. Recent history of abnormal bowel movements, such as diarrhea, loose stools, or constipation,

within 2 weeks of first dose.

16. Had received radiolabeled substances or had been exposed to radiation sources within 12 months of first dose or was likely to receive radiation exposure or radioisotopes within 12 months of first dose such that participation in this study would increase their total exposure beyond the recommended levels considered safe (ie, weighted annual limit recommended by the Commission on Radiological Protection [ICRP] of 3000 mrem).
17. Unable to refrain from or anticipated the use of:
    - Any drug, including prescription and non-prescription medications, herbal remedies, or vitamin supplements within 14 days prior to the first dose and throughout the study. Thyroid hormone replacement medication was permitted if the subject had been on the same stable dose for the immediate 3 months prior to first study drug administration. Acetaminophen (up to 2 g per 24-hour period) was permitted during the study, only after the first dose, if necessary to treat AEs. Milk of magnesia (ie, magnesium hydroxide) (≤60 mL per day) may have been administered approximately on Day 4 (Period 1) or Day 8 (Period 2) to ensure defecation, with agreement between the study investigator and sponsor physician. Additional administration of milk of magnesia may have been needed on other days at discretion of the study investigator and sponsor physician.
    - Any drugs known to be significant inducers of CYP3A enzymes and/or P-glycoprotein (P-gp), including St. John's Wort, within 28 days prior to the first dose and throughout the study. Appropriate sources were consulted to confirm the lack of PK/pharmacodynamic interaction with study drug.
18. Had been on a diet incompatible with the on-study diet, in the opinion of the investigator or designee, within the 30 days prior to the first dose and throughout the study.
19. Donation of blood or significant blood loss within 56 days prior to the first dose.
20. Plasma donation within 7 days prior to the first dose.
21. Participation in another clinical study within 30 days prior to the first dose. The 30-day window was derived from the date of the last blood collection or dose, whichever was later, in the previous study to day 1 of period 1 of the current study.
